# Risky Decisions in a Lottery Task Are Associated with an Increase of Cocaine Use

**DOI:** 10.3389/fpsyg.2016.00640

**Published:** 2016-05-09

**Authors:** Amrei Wittwer, Lea M. Hulka, Hans R. Heinimann, Matthias Vonmoos, Boris B. Quednow

**Affiliations:** ^1^Collegium Helveticum, University of Zurich and Swiss Federal Institute of TechnologyZurich, Switzerland; ^2^Experimental and Clinical Pharmacopsychology, Department of Psychiatry, Psychotherapy, and Psychosomatics, Psychiatric Hospital, University of ZurichZurich, Switzerland; ^3^Center for Addictive Disorders, Department of Psychiatry, Psychotherapy, and Psychosomatics, Psychiatric Hospital, University of ZurichZurich, Switzerland; ^4^Future Resilient Systems, Singapore-ETH CentreSingapore, Singapore; ^5^Department of Environmental Systems Science, ETH ZurichZurich, Switzerland; ^6^Neuroscience Center Zurich, University of Zurich and Swiss Federal Institute of Technology ZurichZurich, Switzerland

**Keywords:** stimulants, impulsivity, gambling, addiction, risky choices, economics, reward, drug dependence

## Abstract

Cocaine use disorder is associated with maladaptive decision-making behavior, which strongly contributes to the harmful consequences of chronic drug use. Prior research has shown that cocaine users exhibit impaired neuropsychological test performances, particularly with regard to attention, learning, and memory but also in executive functions such as decision-making and impulse control. However, to what extent cocaine users show impaired decision-making under risk without feedback has not yet been investigated systematically. Therefore, to examine risk-taking behavior, 31 chronic cocaine users and 26 stimulant-naïve healthy controls who were part of the Zurich Cocaine Cognition Study, performed the Randomized Lottery Task (RALT) with winning lotteries consisting of an uncertain and a certain prospect. Results revealed that risky decisions were associated with male sex, increased cocaine use in the past year, higher cocaine concentrations in the hair, and younger age. In addition, higher levels of cocaine in the hair and cumulative lifetime consumption were associated with risky decisions, whereas potentially confounding factors including cognition and psychiatric symptoms had no significant effect. Taken together, our results indicate that cocaine users who increased their consumption over a period of 1 year show deficits in the processing of risky information accompanied with increased risk-taking. Future research should analyse whether risky decisions could potentially serve as a prognostic marker for cocaine use disorder.

## Introduction

Did cocaine use cause the financial crisis? David Nutt has been heavily criticized for his provocative statement that cocaine-using bankers with their “culture of excitement and drive and more and more and more…got us into this terrible mess” (Anderson, 2013). However, when mechanisms for dealing with uncertain information fail, as in psychiatric conditions such as cocaine use disorder, the results can really be disastrous for the individual (Bolla et al., 1998; Zack and Poulos, 2009) and result in high economic and societal costs (Olesen et al., 2012). Cocaine is the second most used illegal drug in Europe after cannabis (EMCDDA, 2014) and is considered to be the second most harmful drug after heroin (Nutt et al., 2007). Apparently, about 5–6% of cocaine users get addicted within the first year after first use, while 15–16% develop dependency in the long term (Wagner and Anthony, 2002). However, there is yet a lack of longitudinal data and, thus, it remains unclear why some individuals get addicted and others evade an addiction. Although, cocaine is an unselective monoamine reuptake inhibitor (Iversen et al., 2013), its acute rewarding effects have been primarily linked to the inhibition of dopamine transporter function and consequently, increased dopamine levels in the synaptic cleft (Ritz et al., 1987). There is mounting evidence that chronic cocaine consumption is associated with persistent structural and functional adaptive changes in brain areas involved in motivation, reward, judgment, and inhibitory control of behavior (Robinson and Kolb, 2004; Koob and Volkow, 2010). Neuropsychiatric manifestations of cocaine use disorder involve decrements in cognitive domains of attention, working memory, declarative memory, social cognition, and executive functions including decision-making and impulse control (Jovanovski et al., 2005; Nnadi et al., 2005; Verdejo-Garcia et al., 2007; Vonmoos et al., 2013a,b; Hulka et al., 2014; Preller et al., 2014). Specifically, the maladaptive decision-making strategies of cocaine users have been considered to be both: (i) similar to deficits observed in patients with lesions in the orbitofrontal and ventromedial prefrontal cortex; and (ii) an essential attribute of addictive behavior (Bechara, 2005). As effective pharmacological treatment options are currently lacking (Quednow and Herdener, 2016), an adequate characterization of maladaptive decision making as a potential core feature of cocaine addiction could be useful for the development of effective prevention and treatment strategies.

Hence, the effect of chronic drug use on decisions that involve uncertainty, or incomplete knowledge about how choices lead to outcomes, is an important field of addiction research. Indeed, in daily life we continually face trade-offs between options that promise safety and others that carry both potential for jackpot and threat. Sometimes we receive immediate feedback and are able to learn fast and, therefore, improve our decision behavior, whereas other times the outcomes of our decisions are delayed and we only learn slowly over time. In behavioral economics, uncertainty occurs in two domains with different ranges of incompleteness of information: *risk* refers to situations where the expected value of the outcomes are known (e.g., lotteries, insurance); whereas *ambiguity* indicates their likelihood is unknown and is often simply referred to as *uncertainty* (Platt and Huettel, 2008; Shafir, 2008).

Interestingly, most studies on the effect of cocaine use on decision behavior have so far focused on *ambiguous information with feedback*. They have provided evidence that chronic cocaine use is linked to deficits in the processing of reward and punishment contingencies, as measured by the Iowa Gambling Task (IGT; Bechara and Damasio, 2002; Verdejo-Garcia et al., 2007; Kjome et al., 2010). Consequently, it has been suggested that dependent cocaine users fail to incorporate ongoing feedback to guide future behaviors and instead, make impulsive decisions that are based on immediate reward availability (Bechara et al., 2002).

In this study we focussed on decisions under *risky information without feedback* in cocaine users in comparison to an age-, sex-, and verbal intelligence-matched healthy control group. Risky decisions without immediate feedback allow a derivation of solid parameters for the expected value of the outcomes, in contrast to tasks with ambiguous information, such as the IGT. We used a general linear model (GLM) approach enabling a multivariate means of establishing the predictive value of demographic (e.g., sex, age, cognitive function), task-related (e.g., probability), and cocaine- (e.g., dose) related variables concerning risky decisions. Based on a prior study demonstrating deficits in cocaine users in risky decision-making with feedback (Gorini et al., 2014), we hypothesized that cocaine users are more prone to risky decisions and that elevated risk-taking is associated with increased cocaine use.

## Methods

### Participants

All individuals were tested in the 1-year follow-up measurement of the Zurich Cocaine Cognition Study (ZuCo2St; Vonmoos et al., 2013a; Hulka et al., 2014; Vonmoos et al., 2014; Hulka et al., 2015). The recruitment of participants took place at drug prevention and treatment centers, psychiatric hospitals, through advertising in local newspapers, internet platforms, and by word-of-mouth communication (for details see Vonmoos et al., 2013a). Inclusion criteria for all participants were: (1) age between 18 and 60 years, (2) proficiency in German language, (3) no use of prescription drugs affecting the CNS, (4) no current or previous Axis I DSM-IV psychiatric disorder (in cocaine users with exception of cocaine abuse/dependence and/or alcohol and nicotine abuse/dependence, attention deficit hyperactivity disorder, and a history of depression), (5) no neurological disorder or head injury, and (6) no family history of a severe DSM-IV psychiatric disorder such as schizophrenia, bipolar disorder, or obsessive-compulsive disorder. Cocaine users had to meet the DSM-IV criteria for either cocaine abuse or dependence to be included in the study.

Prior to the follow up measurements, participants were instructed to abstain from illegal drugs for ≥3 days and from alcohol ≥24 h. Additionally healthy controls were excluded if they regularly engaged in illegal drug use (>15 occasions) with the exception of occasional cannabis use. Hair samples (6 cm) from all participants were collected at baseline and follow-up and subsequently analyzed with liquid chromatography-mass spectrometry to exclude participants with opioid use and/or pronounced poly-toxic drug use patterns and to objectively characterize cocaine use over the past 6 months (for details see Vonmoos et al., 2013a). In the hair, the concentrations (pg/mg) of cocaine and its main metabolites benzoylecgonine and norcocaine were determined. The total cocaine (coc_tot_) concentration in the hair was then calculated by the following formula according to Hoelzle et al. (2008): coc_tot_ = cocaine + benzoylecgonine + norcocaine. Additionally, the concentration of cocaethylene (coc_eth_) was measured. Coc_eth_ is an ethyl ester of benzoylecgonine formed by the human liver metabolism when cocaine and ethanol co-occur in the blood. Thus, coc_eth_ is a reliable marker for the concomitant use of cocaine and ethanol (Pennings et al., 2002).

Of the 132 participants (79 cocaine users, 53 stimulant-naïve controls) participating in the ZuCo^2^St follow-up, 27 (22 cocaine users, 5 controls) were excluded from the analyses, either because of illegal drug use detected in the hair and not allowed by our exclusion criteria (e.g., opioids or excessive MDMA intake), or because of newly initiated treatment with psychotropic medication (e.g., methylphenidate, antipsychotics, or antidepressants). The risk task was administered in 60 participants (34 cocaine users and 26 controls) only at the follow-up measurement. As one cocaine user had to be excluded from the analysis due to random decision-making and two cocaine users were excluded due to illegal drug use other than cocaine (see above), the final sample consisted of 31 cocaine users and 26 controls. Cocaine users were further divided into groups of (i) 10 persons with a strong increase of cocaine hair concentrations between baseline and 1-year follow-up (*increasers*), (ii) 12 persons with a strong decrease of cocaine hair concentrations (*decreasers*), and (iii) 9 users with largely unchanged cocaine use between both measurement time points (*equal users*). Criteria for increasing and decreasing cocaine use were determined by a combination of absolute and relative changes of cocaine concentration in hair samples between baseline and follow-up. The absolute criterion was based on a shift in cocaine hair concentration of at least 500 pg/mg, according to a commonly accepted cut-off value for reliable detection of cocaine use (Bush, 2008; Cooper et al., 2012). The relative criterion was based on a minimal increase of 20% or a minimal decrease of 10% in coc_tot_ (Vonmoos et al., 2014; Hulka et al., 2015).

The Ethics Committee of the Canton of Zurich approved the study. All participants provided written, informed consent and were compensated for their participation.

### Clinical interviews and questionnaires

At the follow-up, all participants were interviewed with the Structured Clinical Interview for DSM-IV Disorders (SCID-I), which was carried out by a trained psychologist (APA, 1994). Drug use patterns were assessed by means of the Interview for Psychotropic Drug Consumption (IPDC), which has been described in detail elsewhere (Quednow et al., 2004). The brief version of the Cocaine Craving Questionnaire (CCQ) was used to assess current cocaine craving in cocaine users (Sussner et al., 2006). Current symptoms of depression were measured with the Beck Depression Inventory (BDI; Beck et al., 1961). Attention Deficit Hyperactivity Disorder (ADHD) symptoms were assessed with the ADHD Self-Rating Scale (ADHD-SR; Roesler et al., 2004).

### Neuropsychological tests

Participants underwent a broad neuropsychological and social cognitive test battery as well as psychophysiological measurements, which have been described in detail elsewhere (Preller et al., 2013, 2014; Vonmoos et al., 2013a; Hulka et al., 2014). In order to control for the influence of attention, working memory and long-term memory, four representative tests were included into the statistical model as confounding factors: The Letter Number Sequencing Task (LNST) was used to measure verbal working memory function (Wechsler, 1997), for which the dependent variable was the number of correct answers. The Spatial Working Memory task (SWM) strategy score from the Cambridge Neuropsychological Test Automated Battery (CANTAB; Strauss et al., 2006) was used to measure spatial working memory and executive function. The number of total errors tested the capability to retain spatial information and to manipulate remembered items. The Rapid Visual Processing task (RVP) from the CANTAB was administered to assess sustained attention (dependent variable: A′, a signal detection measure of sensitivity that incorporates how well a person is able to detect target sequences). The German version of the Rey Auditory Verbal Learning Test (RAVLT) was administered to assess the verbal declarative memory performance (Helmstaedter et al., 2001), for which the dependent variables were learning performance (Σ trials 1–5) and delayed recall (trial 7).

### Risk task

One of the most common methods to elicit risky decisions without feedback in experimental economics involves the use of binary choice tasks that present winning lotteries in “multiple price lists” (Charness et al., 2013). Participants are presented with a set of binary lotteries where they choose from two options: (A) a lottery with an expected value; and (B) a guaranteed payoff, i.e., a cash amount that can be earned with certainty (Tversky, 1992; Schubert and Brown, 1999; Fehr-Duda, 2006). One multiple price list depicts several decisions with a fixed winning probability and decreasing guaranteed payoff, and therefore when filled out depicts the decision-maker's transition from the safe to the uncertain alternative, or their “indifference point.” Anderson et al. (2007) reported three disadvantages for multiple price lists: First, they are based on internal responses only. Second, subjects often switch back and forth between the choice alternatives, resulting in inconsistent preference elicitation. Third, the multiple price list approach could be susceptible to framing effects, because subjects have a tendency to start in the middle of the table irrespective of the presented values. These disadvantages can be overcome with an experimental task with automatic data capturing that offers the single, binary lotteries in a randomized order. Here we describe such an experimental task, which simulates ecologically valid decisions under risky information with no direct feedback.

At the 1-year- follow-up, all participants performed an interactive risk task, the Randomized Lottery Task (RALT). The task presented a set of 20 binary lotteries to each subject on a computer screen, with each lottery consisting of: option A) a lottery with an expected value characterized by a randomized winning probability of 50 CHF; and option B) a guaranteed payoff that was randomly distributed between zero and the maximum outcome (50 CHF). For example, participants decided between 50 CHF with a probability of 25% *or* a guaranteed payoff of 25 CHF. Different lotteries were automatically generated for each trial: the winning probability and therefore the expected value of the lotteries as well as the guaranteed payoff were fully randomized and equally distributed in the interval between zero and one, in order to allow a comparison between trials and to avoid artifacts.

The RALT started by informing each participant that he/she will play the task for real money. Participants were informed that after completing the RALT, one of the lotteries would be randomly chosen and paid out in real money. A sequence of 20 lotteries appeared on the screen and participants had to make a choice for each of them regarding option A or B. The series of choices was recorded as a sequence consisting of 0 (guaranteed payoff rejected) and 1 (guaranteed payoff preferred) representing the choice behavior (*C*) in our analysis. As the task does not give any feedback, players cannot adapt their strategies over time and the monetary payoff is only revealed at the very end of the game. Therefore, each player acts independently on each decision, which is a prerequisite for the statistical analysis applied. The task additionally recorded the lotteries' so-called uncertainty premium (*up*), which is defined as the difference between the expected value of the lottery minus the value of the guaranteed payoff. Under rationality assumptions, a negative *up* of the lottery would lead to “risk-prone” behavior, whereas a positive *up* would lead to “risk-averse” behavior. The sequence of lotteries was randomized and data were recorded automatically and without immediate feedback. Both the user interface and the data-capture procedure were implemented with Visual Basic for Application (VBA) and a spreadsheet program (Microsoft EXCEL 2007).

### Definition of variables

Following Montgomery (1999), we defined four types of variables (Table 1), namely the response variable, covariates, factors, and nuisance covariates: risk behavior *C*, captured by the RALT, was our response variable that we aimed to explain with covariates (cocaine consumption, lotteries *up*, and age parameters; measured continuously on a scale), which are modified by factors (sex, cocaine user group, and cocaine craving; discrete classes) and nuisance variables (ADHD symptoms, depressive symptoms, attention, verbal, and spatial working memory, as well as verbal declarative memory). Prior findings have provided compelling evidence that dependent (Jovanovski et al., 2005; Vonmoos et al., 2013a) and to some extent also recreational cocaine users (Soar et al., 2012; Vonmoos et al., 2013a) exhibit broad neurocognitive deficits in attention, verbal, and spatial working memory, as well as verbal declarative memory. Moreover, cocaine users are more likely to suffer from depression and ADHD (Vonmoos et al., 2013a). Therefore, in order to control for potentially confounding effects of these nuisance variables on risk-taking behavior, we included them in the GLM. Table 1 provides additional description and the acquisition method.

**Table 1:** **Definition of variables and acquisition methods**.

**Variable type**	**Symbol**	**Description of variable**	**Acquisition method**
Response	*C*	Binary variable 0 choice of the uncertain alternative 1 choice of the safe alternative	Automatically recorded by the RALT
Covariates	*up*	Uncertainty premium of the safe alternative of a lottery Expected value (x; p)–cash value (y; 1)	Automatically recorded by the RALT
	*p*	Probability of the uncertain alternative of a lottery	Automatically recorded by the RALT
	coc_lt_	Lifetime amount of cocaine (g)	Drug interview
	coc_eth_	Cocaethylene in the hair (pg/mg)	LC-MS/MS analysis of the hair
	coc_tot_	Sum of cocaine, and its metabolites benzoylecgonine and norcocaine in the hair (pg/mg)	LC-MS/MS analysis of the hair
	age	Biological age (y)	Questionnaire
Factor	SEX	Biological aspects of femaleness/maleness	Questionnaire
	INC	Cocaine concentration measured in hair Factor 10 controls (no cocaine use) 20 increasing cocaine use 30 decreasing cocaine use 40 steady cocaine use	LC-MS/MS analysis of the hair
	CCQ	Craving for cocaine (score 0–70)	Cocaine Craving Questionnaire
Nuisance covariates	ADHD	Attention deficit hyperactivity disorder symptoms (score 0–54)	ADHD Questionnaire
	BDI	Symptoms of depression (score 0–63)	Beck Depression Inventory
	RVP	Sustained attention (score 0–1)	Rapid Visual Processing task CANTAB
	LNST	Verbal working memory (score 0–24)	Letter Number Sequencing Task
	RAVLTsum	Verbal declarative memory performance (Σtrials 1–5, score 0–75)	Rey Auditory Verbal Learning Test
	RAVLT7	Verbal declarative memory, delayed recall (score 0–15)	Rey Auditory Verbal Learning Test
	SWM	Spatial Working Memory/Executive function (score 1–37)	Spatial Working Memory task CANTAB

*LC-MS/MS, liquid chromatography-tandem mass spectrometry*.

### Statistical analysis

The statistical analysis was based on generalized linear models (McCullagh and Nelder, 1989) using the statistical software R, aiming to identify a model that best explains the choice behavior (C) with the explanatory variables (Table 1). Since the response variable is binary, a logit transformation was used, assuming linearity of all the explanatory variables in the logit space (Equation 1):

(1) $$\begin{array}{l} g\left(n\right)=\mu +up+p+SEX_{i}+\left[COC_{it}+\ldots \right]+\\ \left[CCQ+BZT+RAVLT_{sum}\right]+\varepsilon_{ij} \end{array}$$

where η, linear predictor; *g*(), link function [logit]; μ, overall mean; *up*, lotteries uncertainty premium [CHF, risk task input]; *p*, probability of the uncertain alternative; SEX_j_, sex [factor, i = m, f]; [coc_lt_ + …], cocaine use parameters [see Table 1]; [CCQ,…], nuisance due to craving and cognitive detriment [see Table 1]; ε_ij_, error term, binomial distributed.

Linear and generalized linear models assume a lack of multicollinearity in the explanatory variables, which was analyzed with correlation diagnostics (R *cor* function). The model consists of three classes of explanatory variables: (1) the utility parameters (mean value μ, *up*, sex), (2) cocaine use parameters (estimated lifetime cocaine use [coc_lt_], CCQ score, hair parameters coc_eth_ and coc_tot_,), and (3) cognitive and clinical covariates (ADHD, BDI, RVP, LNST, RAVLT_sum_, RAVLT_7_, SWM). As the number of observations in each class of sex (male, female) was unbalanced, which is typical for observational studies, a weighted squares of means analysis (Searle, 1987) was necessary.

A log transformation of the cocaine use parameters (coc_lt_, coc_eth_, coc_tot_, CCQ) was calculated in order to eliminate the heavy skewness of the data distribution and increase the homogeneity of the residual variance. To identify the subset of variables with the highest explanatory power the following strategy was used: first, in order to depict the power of single effects, a type II deviance analysis was calculated, which is an analog to the variance analysis in linear models (Fox et al., 2011). A type II analysis compares the full model with all variables with (full-1) models that sequentially leave-one-out of all variables (Langsrud, 2003). Strategies for developing models and for the selection of the variables also recommend investigating the effect of first-order interactions (e.g., sex^*^up). Second, an applied standard procedure was used to estimate the values and standard errors of parameters (**Tables 5**, **6**), including backwards stepwise elimination based on the Akaike Information Criterion (R *stepAIC function*). Since this procedure may risk over-fitting models, we used variance inflation metrics (R *vif function*) to estimate the severity of multicollinearity and eliminate collinear explanatory variables. Third, we performed model diagnostics, such as studentized residuals, hat values and cook's distance to assess if error distributions were homogeneous and if there were influential or leveraging observations (R *influencePlot*). The outlier analysis was performed with the R *outlierTest* function.

**Figure 1:**
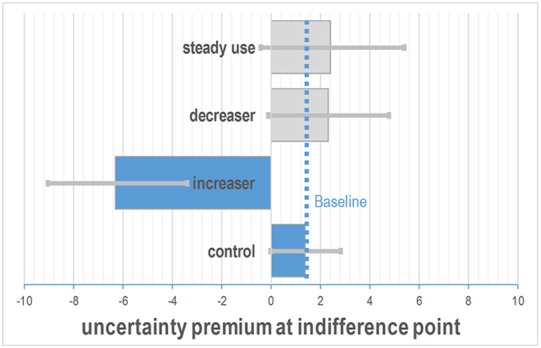
**Difference in indifference points between the controls and the groups of cocaine users**. Increasers had negative indifference points indicating increased risk-taking behavior. The dotted line depicts the indifference point of the control group as the baseline condition. The estimated uncertainty premium was calculated for the four increaser classes with SEX = female; age = 30, *p* = 0.5. Bars depict estimated 95% confidence intervals.

**Figure 2:**
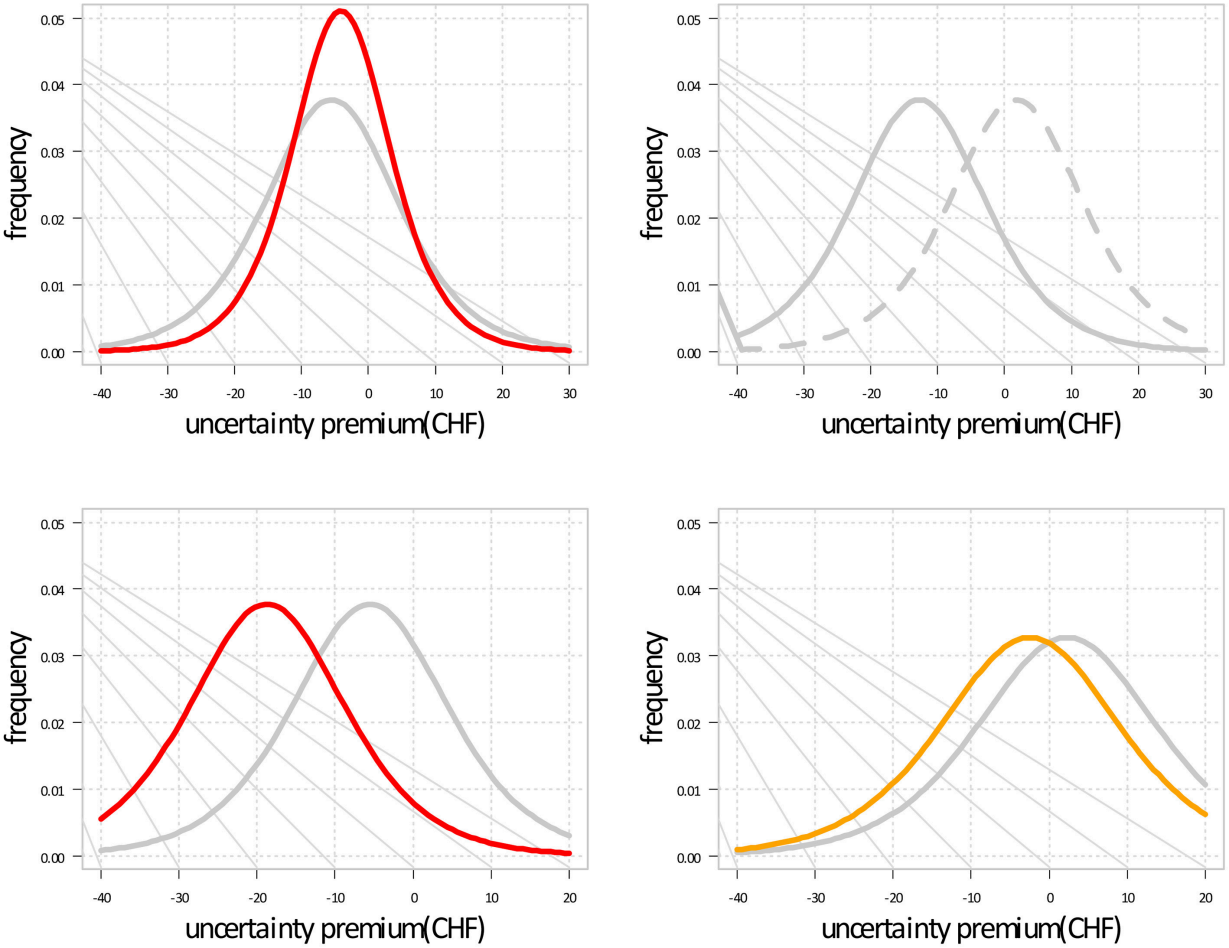
**Effect of sex, winning probability, increasing cocaine use, and cocaine in the hair on the participants' choice depicted by the distribution of the indifference points**. Negative uncertainty premiums represent risk-prone behavior, while positive values indicate risk-averse behavior. [upper left] male (red) behavior shows smaller variability; [upper right] the change of the winning probability from 0.05 to 0.95 (pointed line) results in a shift toward risk-aversion; [lower left] increasing cocaine use (red) results in a shift toward risk-proneness; [lower right] high cocaine concentration in the hair (yellow) at the 95% quantile of all individuals results in a slight shift toward risk-proneness.

As a risk measure derived from the choice behavior *C*, we calculated the indifference points, where the probabilities of choosing options A or B were both 0.5. This approach has been used widely in experimental economics. We calculated indifference points for all groups (*increasers, decreasers, equal users, control)* based on cocaine toxicological hair analyses (Figure 1). For each of the indifference points, confidence intervals were estimated in logit space and transformed back to the observation space. As the indifference points depend on the *up* of the lotteries, they were depicted as a probability distribution, our additional risk metric (Figure 2).

## Results

### Demographic variables and drug use

Table 2 presents means, standard deviations and *t*-test statistics for demographic variables and drug use patterns. Healthy controls and cocaine users did not differ with regard to age and verbal IQ. In contrast, cocaine users had significantly fewer years of education and higher depression and ADHD scores compared to healthy controls. There was also a trend toward a greater number of males in the cocaine users than in the control group. Cocaine users had an average lifetime cocaine consumption of 744 g and hair toxicology analyses confirmed that cocaine was the primary drug of choice.

**Table 2:** **Demographic data, drug use patterns, neuropsychological and risk tasks**.

	**Stimulant-naïve controls (*n* = 26)**	**Cocaine users (*n* = 31)**	***t***	***df***	***p***
**DEMOGRAPHY**					
Age	31.62 (9.14)	30.81 (7.87)	0.36	55	0.72
Sex (number/%)	10/16 38/62%	5/26 16/84%	3.64^‡^	1	0.06
Verbal IQ	109.58 (11.64)	106.32 (9.28)	1.18	55	0.25
Years of education	11.10 (1.80)	10.16 (1.49)	2.11	55	**0.04**
BDI	1.85 (2.33)	8.35 (9.50)	–3.69	55	**0.00**
ADHD-SR score	7.69 (4.92)	14.97 (8.88)	–3.90	55	**0.00**
**COCAINE USE**					
Lifetime (g)	–	744.04 (927.09)	–	–	–
Coc_tot_ (pg/mg)	–	14120 (37769)	–	–	–
Coc_eth_ (pg/mg)	–	818 (1750)	–	–	–
CCQ	–	18.06 (8.84)	–	–	–
**NEUROPSYCHOLOGICAL TESTS**					
RVP A′	0.94 (0.04)	0.92 (0.04)	1.78	55	0.08
LNST	15.19 (2.79)	14.61 (3.03)	0.75	55	0.46
RAVLT7	13.92 (1.73)	12.65 (2.51)	2.24	54	**0.03**
RAVLT_sum_	66.56 (5.46)	60.65 (9.34)	2.96	54	**0.01**
SWM	30.00 (6.81)	32.48 (4.52)	–1.59	55	0.12
**RISK TASK (RALT)**					
*C*	0.39(0.49)	0.45(0.49)	–2.09	1123	**0.04**
*up*	–2.14(9.4)	–1.38(9.25)	–1.39	1106	0.16
*p*	0.49(0.26)	0.47(0.26)	1.16	1102	0.25

*BDI, Beck Depression Inventory; ADHD-SR, Attention-Deficit Hyperactivity Disorder Self-Rating Scale; C, choice behavior; Coctot, cocaine + benzoylecgonine + norcocaine concentration in hair; Coceth, ethylcocaine in hair sample; CCQ, Cocaine Craving Questionnaire; p, probability of the uncertain alternative, RVP A′, Rapid Visual Processing task A′; LNST, Letter Number Sequencing Task; RAVLT sum, Rey Auditory Verbal Learning Test (Σtrials 1-5); RAVLT7, Rey Auditory Verbal Learning Test (trial 7); SWM, Spatial Working Memory; up, uncertainty premium. Significant p-values are shown in bold. Statistical tests: Independent t-tests for continuous data and ‡chi^2^ for frequency data*.

### Neuropsychological tests and risk task

In order to control for general neuropsychological performance with regard to preferences in the RALT risk task, verbal working memory function (LNST), spatial working memory and executive function (SWM), sustained attention (RVP'A), and verbal declarative memory performance were measured (RAVLT; Table 2). Healthy controls and cocaine users did not significantly differ with regard to the RVP A′, LNST, and SWM, but cocaine users performed significantly worse in both RAVLT parameters, reflecting impaired verbal declarative learning capacity and reduced delayed verbal recall. With regard to the RALT, cocaine users had significantly higher *C* values, indicating that cocaine users on average preferred the risky alternative.

### Task correlations

The correlation-matrix identifies collinearities within a data set, which is a prerequisite for proper GLM-model selection. Table 3 depicts the correlation coefficients. In order to avoid accumulation of alpha error, the significance threshold for correlations was set at *p* < 0.01. Decision behavior *C* was significantly correlated with the *up* of the lotteries (*r* = 0.46). Moreover, coc_lt_ was negatively correlated with sustained attention (RVP), working memory (LNST), verbal learning capacity (RAVLT_sum), and verbal long-term memory (RAVLT7). Additionally, increased coc_eth_ in hair was correlated with reduced sustained attention, suggesting that the pronounced concomitant use of alcohol and cocaine is considerably worse for cognitive functioning, as shown previously (Bolla et al., 2000). These findings recapitulate the previously-reported correlations between cocaine use and cognitive impairment in this sample (Vonmoos et al., 2013a, 2014). Finally, higher age was correlated with lower verbal working memory, while several additional cognitive parameters were intercorrelated (e.g., worse sustained attention was associated with decreased verbal declarative memory and an increased, i.e., less efficient, SWM strategy score).

**Table 3:** **Correlation-matrix with *r*-values for cardinal variables**.

	***C***	***up***	***p***	**coc_lt_**	**coc_eth_**	**coc_tot_**	**Age**	**RVP**	**LNST**	**RAVLT_sum_**		**RAVLT**_**7**_		**SWM**
*C*	1.00													
*up (lot)*	**0.46**	1.00												
*p*	−0.26	0.07	1.00											
coc_lt_	0.01	0.04	−0.07	1.00										
coc_eth_	−0.11	0.04	0.00	**0.40**	1.00									
coc_tot_	−0.07	0.03	−0.02	**0.48**	**0.87**	1.00								
Age	−0.10	0.01	0.00	0.27	0.20	0.25	1.00							
RVP	0.06	−0.03	0.03	−**0.44**	−**0.40**	−0.32	−0.30	1.00						
LNST	0.02	0.04	0.04	−**0.33**	−0.21	−0.32	−**0.34**	0.33	1.00					
RAVLT_*sum*_	0.07	0.00	0.01	−**0.42**	−0.23	−0.27	−0.18	**0.49**	**0.34**	1.00				
RAVLT_7_	0.07	−0.01	0.03	−**0.43**	−0.24	−0.30	−0.15	**0.42**	0.31	**0.91**	1.00			
SWM	−0.02	−0.01	−0.04	0.24	0.26	0.32	0.10	−**0.43**	−0.36	−0.28	−0.18		1.00	

*BDI, Beck Depression Inventory; ADHD, attention-deficit hyperactivity disorder; C, choice behavior; Coctot, cocaine, benzoylecgonine, norcocaine in hair sample; Coceth, ethylcocaine in hair sample; CCQ, cocaine craving questionnaire; p, probability of the uncertain alternative, RVP A′, Rapid Visual Processing task A′; LNST, Letter Number Sequencing Task; RAVLT sum, Rey Auditory Verbal Learning Test (Σtrials 1-5); RAVLT7, Rey Auditory Verbal Learning Test (trial 7); SWM, Spatial Working Memory; up, uncertainty premium. Significant correlations (p < 0.01) are shown in bold*.

The three parameters (coc_lt_, coc_eth_, coc_tot_) were heavy-tailed: about 90% of the coc_eth_, coc_tot_ data are located in the lowest 10% of the data range, whereas this amount is close to 75% for coc_lt_. Therefore, a log-transformation of these parameters was used in further calculations to eliminate the heavy skewness of the data distribution and increase the homogeneity of the residual variance.

### Analysis of deviance

The analysis of variance likelihood ratio test of the RALT parameter *C* including all 15 explanatory variables was significant for seven variables (Table 4). Explanatory variables with a stronger effect on risk-taking *C* had a higher likelihood ratio, measured with the likelihood chi-square. Concerning first-order interactions, the interaction of *up* and sex was chosen in the final model, as the partial deviance of this interaction term is higher than the sum of partial deviance of the two single variables.

**Table 4:** **Analysis of deviance table (type II analysis)**.

	**LR Chi^2^**	***df***	***p*(>Chisq)**
*p*	79.8	1	**0.01**
CCQ	0.02	1	0.89
BDI	1.22	1	0.26
ADHD	0.01	1	0.98
Age	5.46	1	**0.02**
coc_eth_	44.33	1	**0.01**
coc_lt_	1.70	1	0.19
coc_tot_	14.04	1	**0.01**
INC	61.52	4	**0.01**
RVP A′	2.11	1	0.15
LNST	2.54	1	0.11
RAVLT_sum_	2.85	1	0.09
RAVLT7	0.36	1	0.53
SWM	0.16	1	0.69
up^*^SEX	364.31	2	**0.01**

*Deviance measures the explanatory power of the model components. The interaction of SEX and risk premium had the biggest influence, followed by the probability of the lottery p, INC, coc_eth_, and coc_tot_. BDI, Beck Depression Inventory; ADHD, attention-deficit hyperactivity disorder; Coctot, cocaine, benzoylecgonine, norcocaine in hair sample; Coc_eth_, ethylcocaine in hair sample; CCQ, cocaine craving questionnaire; INC, factor group, p, probability of the uncertain alternative, RVP A′, Rapid Visual Processing task A′; LNST, Letter Number Sequencing Task; RAVLT sum, Rey Auditory Verbal Learning Test (Σtrials 1–5); RAVLT7, Rey Auditory Verbal Learning Test (trial 7); SWM, Spatial Working Memory; up, uncertainty premium. Significant p-values are shown in bold*.

The interaction of the lotteries *up* and sex had the strongest effect, followed by the lotteries winning probability, increase in cocaine use, the cocaine concentration in the hair and age. A model with 15 parameters bears the risk of over-fitting and variance inflation. The variance inflation test yielded five variables with a variance inflation factor higher than 5, which indicated the presence of collinearities that were omitted in the next step.

### Model based on changing cocaine use patterns within 1 year

The stepwise selection procedure based on the Akaike Information Criterion (AIC) resulted in a model with 5 parameters only (Table 5): the interaction of the lotteries *up* and sex, the lotteries winning probability, cocaine use pattern and age. Since cocaine use pattern is a factor with 4 levels, a single parameter was estimated for each level, whereas the standard level equals the intercept. Only users with an increase of cocaine use (level 20) were significantly different from the controls (level 10).

**Table 5:** **Parameter estimation for a model based on cocaine use patterns**.

	**Estimate**	**SE**	***z*-value**	**Significance**
(Intercept)	2.51	0.36	6.87	^***^
*p*	−2.41	0.27	−8.75	^***^
Age	−0.05	0.01	−5.11	^***^
Group (increasers)	1.08	0.22	4.97	^***^
Group (decreasers)	−0.13	0.20	−0.65	
Group (steady use)	−0.15	0.23	−0.64	
Group (control)	−0.15	0.52	−0.29	
up^*^SEXf	0.14	0.01	9.81	^***^
up^*^SEXm	0.19	0.02	10.57	^***^

*p, probability of the uncertain alternative; SEXf, female sex, SEXm, male sex; up, uncertainty premium*.

*** *p < 0.001*.

Figure 1 shows that the estimated indifference points of the *increasers* were negative, indicating strong risk-taking behavior, whereas controls, steady users and *decreasers* had a positive risk premium, indicating a more risk adverse behavior. The depicted indifference points correspond to the lotteries *up* with the highest probability of choice.

Figure 2 depicts the effect of the explanatory variables on the probability distribution of the indifference point, depending on the *up* of the lotteries. Increasing cocaine use and high cocaine concentrations in the hair were associated with participants' negative *up* indicating risk-prone behavior. Male sex was associated with a smaller variability in the distribution of the indifference points.

### Model based on cocaine hair concentrations

The stepwise selection procedure based on AIC resulted in a 4 parameter model (Table 6) consisting of the interaction of the lotteries *up* and sex, the lotteries winning probability, and the logarithm of average of coc_tot_ in the hair. Coc_tot_ was automatically selected because it showed a higher effect than cocaethylene and cumulative lifetime consumption of cocaine on risk taking. All the parameters were significant at *p* < 0.001 and the test for variance inflation resulted in values close to one, indicating no co-linearity.

**Table 6:** **Parameter estimation for a model based on cocaine in the hair**.

	**Estimate**	**SE**	***z* value**	
(Intercept)	0.88	0.16	5.59	^***^
*p*	−2.36	0.27	−8.82	^***^
Log (coc_tot_ + 1)	0.091	0.027	3.38	^***^
up^*^SEXf	0.13	0.014	9.32	^***^
up^*^SEXm	0.18	0.017	10.68	^***^

*Coc_tot_, cocaine, benzoylecgonine, norcocaine in hair sample; p, probability of the uncertain alternative; SEXf, female sex, SEXm, male sex; up, uncertainty premium*.

*** *p < 0.001*.

Figure 2 (lower right) depicts the effect of cocaine concentrations in the hair on the probability distribution of the indifference point and indicates that high cocaine concentrations in the hair were associated with participants' negative *up*, indicating risk-prone behavior.

## Discussion

In this study, we report on differences in non-social decision-making without immediate feedback in cocaine users in comparison with a control group. Careful psychiatric diagnostic procedures ensured that cocaine users had few psychiatric co-morbidities and detailed toxicological hair analyses showed relatively sparse polysubstance use. Importantly, we used the GLM analysis approach as a technique to identify demographic, task-related, and cocaine-related variables with the highest explanatory power for risk-taking. Our study yielded the following major findings: (I) Cocaine users, as a group, are more prone to risky decisions in a lottery task in comparison to a matched control group without regular drug use. (II) Risky decisions are associated with male sex, self-reported increase in cocaine use, higher amounts of cocaine in the hair and younger age. More specifically, (III) *increasers* made significantly more risky decisions in the lotteries than the other groups as they chose less favorable options with a higher probability for losing in the lottery task, depicted by a negative indifference point. Moreover, (IV) higher concentrations of cocaine in the hair and cumulative lifetime consumption of cocaine across all user groups were associated with risky decisions, whereas potentially confounding factors like attention, working memory, long-term memory, symptoms of depression and ADHD had no significant effect. Taken together, our results indicate that cocaine users who increased their consumption in the last 12 months show deficits in the processing of risky information and exhibit increased risk-taking in comparison to the other groups of cocaine users and controls.

To our knowledge, the effect of cocaine use on *risky decisions without feedback* has not yet been investigated with an experimental economic analysis approach. One study examined *risky decisions with immediate feedback* in the game of dice task that presents choices between different lotteries with known expected utility. In congruence with our results, cocaine users made a significantly reduced number of safe bets although they only gambled with hypothetical monetary gains (Gorini et al., 2014).

Moreover, several previous studies have shown that cocaine users exhibit disadvantageous decision-making in *ambiguous decisions with immediate feedback* that require adequate processing of reward and punishment contingencies as measured by the IGT (Bechara et al., 2002; Verdejo-Garcia et al., 2007; Vadhan et al., 2009; Kjome et al., 2010; Balconi et al., 2014) and by the Balloon Analogue Risk Task (Canavan et al., 2014): However, some studies also report no significant effect of cocaine use on performance in either the IGT (Bolla et al., 2003; Hulka et al., 2014) or the Balloon Analogue Risk Task (Gorini et al., 2014). The reasons for these inconsistent results remain unclear. It has been suggested that dependent cocaine users fail to incorporate ongoing feedback to guide future behaviors, make impulsive decisions that are based on immediate reward (Bechara et al., 2002) and fail to learn from repeated mistakes because of an insensitivity to future consequences (Bechara, 2001).

Our results indicate that risky decisions in cocaine users can also be observed without feedback, indicating an additional and more basic mechanism than the failure to learn from immediate mistakes. Zack and Poulos (2009) proposed that “…the placing of the bet, execution of the play, and anticipation of its outcome may induce a subjective state (suspense/thrill) quite apart from the outcome itself (win/lose). Thus, in gambling, it is possible to subjectively *like* the state of *wanting*. This is not unique to gambling; it is seen in seduction or striptease, as well as in activities like hunting. In each case, the subject enjoys the state of pursuit *per se*. […]. One likely subjective correlate of such pursuit is arousal.” In the present study participants received no feedback on the outcome of their decisions, however, they gambled with real money as they were informed that they would receive a monetary gain at the end of the study. Our results indicate that even without the feedback of “you win!” or “you lose!” cocaine users and controls make significantly different risky decisions. Regarding possible basic mechanisms of this finding, neurophysiological studies in monkeys reveal that dopamine neurons show phasic activations related predominantly, but not exclusively, to feedback (reward and punishment), but also without feedback to risk, expectancy of reward, and the salience of the stimulus: the activations increase with reward value, probability and their multiplied product, expected value (Schultz, 2010). Dopamine has been widely implicated in reinforcement, reward, and pleasure (Schultz, 2015). In addition, Panksepp (1998) characterized dopamine as the “seeking-system” and similarly Berridge (2007) have argued that dopamine is more strongly involved in the “wanting”—i.e., the moment before and without the feedback—than in the “liking” of a result. Dopamine therefore drives the ability to recognize a stimulus as a signal for future reward and to elicit approach behavior even without direct reward. Here, we focused on this “wanting” aspect of gambling behavior in cocaine users. Interestingly, dopamine plays a similar role in pathological gambling to its role in psychostimulant addiction: chronic exposure to both gambling and psychostimulants is believed to induce profound and long lasting changes in brain function, where sensitization of dopamine pathways are considered to play a critical underlying role in the pathological effect (Robinson and Berridge, 2001). Importantly, Zack and Poulos (2009) highlighted that the signals for uncertain reward in gambling lead to dynamic changes in dopamine release, much like that induced by psychostimulant drugs. They hypothesize that these changes in dopamine release may reinforce gambling behavior regardless of its outcome. Our results may be in accordance with this hypothesis. Following this line of thought, reward-predicting stimuli may induce a pseudo-cocaine-state of “wanting” in cocaine users leading to risk-prone behavior and the ignorance of the long-term negative consequences, due to sensitization of dopamine pathways.

It has been suggested that hypothetical monetary gains might not always offer enough incentive for gambling behavior in cocaine users, therefore—in contrast to most previous studies (Bechara and Damasio, 2002; Kjome et al., 2010; Balconi et al., 2014; Canavan et al., 2014; Gorini et al., 2014)—our participants gambled with real money. However, rewards have been shown to lose some of their value when they are delayed, even though their objective reward value is the same (Ainslie, 1975). This effect might be increased in cocaine users, since they display a highly stable preference for smaller, immediate rewards compared to larger but delayed rewards (Hulka et al., 2014, 2015). However, our results indicate that the expected reward of a monetary payment at the end of the study was large enough to motivate “wanting”—behavior in cocaine users.

There are some limitations inherent to the present study. Firstly, it is impossible to substantiate whether the differences in risky decisions in cocaine users are due to a predisposition, to cocaine-induced neuroplasticity, or to an interaction of both, given that we assessed risk-taking behavior only cross-sectionally and only at the follow-up of the ZuCo^2^St. Secondly, our results indicate that an increase in substance use is associated with a high level of risk-taking. Importantly, these results have no prognostic value so far as risk-taking was measured only at the follow-up study. Thus, it is possible that risk-taking did increase in line with cocaine use and therefore might not be valid as a prognostic marker for the development of addiction. However, this finding is interesting for future research in the context of the question of why some users get addicted while others don't, but it remains to be shown whether risk-taking could be a predictor for the development of an addiction. Thirdly, the group size was relatively modest, mainly due to our strict inclusion criteria. However, we have measured relatively pure cocaine users with very little psychiatric and no medical comorbidities, which is also an advantage. Finally, stepwise regression procedures of the kind we employed have been criticized (Henderson and Denison, 1989; Sribney, 2011). Sribney (2011) gave a helpful overview on the problems of stepwise regression analyses—e.g., in the presence of collinearity or outliers. Importantly, a GLM analysis is only a model for reality and will never depict cause/effect relationships, only possible (statistically significant) associations.

In conclusion, our results indicate that even in risky decisions without direct feedback cocaine users differ in their behavior, exhibiting risk-proneness. Interestingly, users who have increased their consumption over a period of 1 year show deficits in the processing of risky information. Considering that real world financial risks do often come with an expected value and delayed feedback this evidence strengthens the point that cocaine use could add to mismanagement and should be avoided by decision makers. Importantly, Bechara (2005) has proposed that drug users with impaired decision-making might be more vulnerable to embarking on a downward-spiraling path, because poor decision-making leads to addiction. Following this argument, future prospective studies should analyse whether neurocognitive development depicted by risky decisions could serve as a marker for addictive disorders and success of treatment.

## Author contributions

AW wrote the first draft of the manuscript and contributed to the cooperation and design of the study. The risk task was designed by HH and AW. LH and MV obtained the data. The data were analyzed by HRH as well as AW and LH. AW, LH, MV, and HH were involved in the revision of the manuscript. BQ designed the study, interpreted the results, wrote and revised the manuscript, and bears responsibility for data acquisition and analyses.

### Conflict of interest statement

The authors declare that the research was conducted in the absence of any commercial or financial relationships that could be construed as a potential conflict of interest.

## References

[B1] Ainslie G. (1975). Specious reward: a behavioral theory of impulsiveness and impulse control. Psychol. Bull. 82, 463–496. 10.1037/h0076860 1099599

[B2] Anderson G. (2013). Did Cocaine Use by Bankers Cause the Global Financial Crisis? The Guardian [Online]. Available online at: http://www.theguardian.com/business/shortcuts/2013/apr/15/cocaine-bankers-global-financial-crisis

[B3] Anderson S. Harrison G. W. Lau M. I. Rutstrom E. E. (2007). Valuation using multiple price list formats. Appl. Econ. 39, 675–682. 10.1080/00036840500462046

[B4] APA (1994). American Psychological Association. Diagnostic and Statistical Manual of Mental Disorders: DSM-IV. Washington, DC: American Psychiatric Association (APA).

[B5] Balconi M. Finocchiaro R. Campanella S. (2014). Reward sensitivity, decisional bias, and metacognitive deficits in cocaine drug addiction. J. Addict. Med. 8, 399–406. 10.1097/ADM.0000000000000065 25303980

[B6] Bechara A. (2001). Neurobiology of decision-making: risk and reward. Semin. Clin. Neuropsychiatry 6, 205–216. 10.1053/scnp.2001.22927 11447572

[B7] Bechara A. (2005). Decision making, impulse control and loss of willpower to resist drugs: a neurocognitive perspective. Nat. Neurosci. 8, 1458–1463. 10.1038/nn1584 16251988

[B8] Bechara A. Damasio H. (2002). Decision-making and addiction (part I): impaired activation of somatic states in substance dependent individuals when pondering decisions with negative future consequences. Neuropsychologia 40, 1675–1689. 10.1016/S0028-3932(02)00015-5 11992656

[B9] Bechara A. Dolan S. Hindes A. (2002). Decision-making and addiction (part II): myopia for the future or hypersensitivity to reward? Neuropsychologia 40, 1690–1705. 10.1016/S0028-3932(02)00016-7 11992657

[B10] Beck A. T. Ward C. H. Mendelson M. Mock J. Erbaugh J. (1961). An inventory for measuring depression. Arch. Gen. Psychiatry 4, 561–571. 10.1001/archpsyc.1961.01710120031004 13688369

[B11] Berridge K. C. (2007). The debate over dopamine's role in reward: the case for incentive salience. Psychopharmacology (Berl). 191, 391–431. 10.1007/s00213-006-0578-x 17072591

[B12] Bolla K. I. Cadet J. L. London E. D. (1998). The neuropsychiatry of chronic cocaine abuse. J. Neuropsychiatry Clin. Neurosci. 10, 280–289. 10.1176/jnp.10.3.280 9706535

[B13] Bolla K. I. Eldreth D. A. London E. D. Kiehl K. A. Mouratidis M. Contoreggi C.. (2003). Orbitofrontal cortex dysfunction in abstinent cocaine abusers performing a decision-making task. Neuroimage 19, 1085–1094. 10.1016/S1053-8119(03)00113-7 12880834 PMC2767245

[B14] Bolla K. I. Funderburk F. R. Cadet J. L. (2000). Differential effects of cocaine and cocaine plus alcohol on neurocognitive performance. Neurology 54, 2285–2292. 10.1212/WNL.54.12.2285 10881254

[B15] Bush D. M. (2008). The U.S. Mandatory guidelines for federal workplace drug testing programs: current status and future considerations. Forensic Sci. Int. 174, 111–119. 10.1016/j.forsciint.2007.03.008 17434274

[B16] Canavan S. V. Forselius E. L. Bessette A. J. Morgan P. T. (2014). Preliminary evidence for normalization of risk taking by modafinil in chronic cocaine users. Addict. Behav. 39, 1057–1061. 10.1016/j.addbeh.2014.02.015 24642345 PMC4026273

[B17] Charness G. Uri G. Alex I. (2013). Experimental methods: eliciting risk preferences. J. Econ. Behav. Organ. 87, 43–51. 10.1016/j.jebo.2012.12.023

[B18] Cooper G. A. Kronstrand R. Kintz P. Society of Hair T. (2012). Society of Hair Testing guidelines for drug testing in hair. Forensic Sci. Int. 218, 20–24. 10.1016/j.forsciint.2011.10.024 22088946

[B19] EMCDDA (2014). European Monitoring Centre for Drugs and Drug Addiction. European Drug Report 2014. Trends and developments. Luxembourg: Publications Office of the European Union.

[B20] Fehr-Duda H. (2006). Gender, financial risks, and probability weights. Theory Decis. 60, 283–313. 10.1007/s11238-005-4590-0

[B21] Fox E. Sudderth E. B. Jordan M. I. Willsky A. S. (2011). Bayesian nonparametric inference of switching dynamic linear models. IEEE Trans. Signal Process. 59, 1569–1585. 10.1109/TSP.2010.2102756

[B22] Gorini A. Lucchiari C. Russell-Edu W. Pravettoni G. (2014). Modulation of risky choices in recently abstinent dependent cocaine users: a transcranial direct-current stimulation study. Front. Hum. Neurosci. 8:661. 10.3389/fnhum.2014.00661 25221496 PMC4145470

[B23] Helmstaedter C. Lendt M. Lux S. (2001). Verbaler Lern- und Merkfähigkeitstest. Göttingen: Beltz.

[B24] Henderson D. Denison D. R. (1989). Stepwise regression in social and psychological research. Psychol. Rep. 64, 251–257. 10.2466/pr0.1989.64.1.251

[B25] Hoelzle C. Scheutler F. Uhl M. Sachs H. Thieme D. (2008). Application of discriminant analysis to differentiate between incorporation of cocaine and its congeners into hair and contamination. Forensic Sci. Int. 176, 13–18. 10.1016/j.forsciint.2007.07.020 18063333

[B26] Hulka L. M. Eisenegger C. Preller K. H. Vonmoos M. Jenni D. Bendrick K.. (2014). Altered social and non-social decision-making in recreational and dependent cocaine users. Psychol. Med. 44, 1015–1028. 10.1017/S0033291713001839 23870112

[B27] Hulka L. M. Vonmoos M. Preller K. H. Baumgartner M. R. Seifritz E. Gamma A.. (2015). Changes in cocaine consumption are associated with fluctuations in self-reported impulsivity and gambling decision-making. Psychol. Med. 45, 3097–3110. 10.1017/S0033291715001063 26081043

[B28] Iversen L. Gibbons S. Treble R. Setola V. Huang X. P. Roth B. L. (2013). Neurochemical profiles of some novel psychoactive substances. Eur. J. Pharmacol. 700, 147–151. 10.1016/j.ejphar.2012.12.006 23261499 PMC3582025

[B29] Jovanovski D. Erb S. Zakzanis K. K. (2005). Neurocognitive deficits in cocaine users: a quantitative review of the evidence. J. Clin. Exp. Neuropsychol. 27, 189–204. 10.1080/13803390490515694 15903150

[B30] Kjome K. L. Lane S. D. Schmitz J. M. Green C. Ma L. Prasla I.. (2010). Relationship between impulsivity and decision making in cocaine dependence. Psychiatry Res. 178, 299–304. 10.1016/j.psychres.2009.11.024 20478631 PMC2904056

[B31] Koob G. F. Volkow N. D. (2010). Neurocircuitry of addiction. Neuropsychopharmacology 35, 217–238. 10.1038/npp.2009.110 19710631 PMC2805560

[B32] Langsrud Y. (2003). ANOVA for unbalanced data: use Type II instead of Type III sums of squares. Stat. Comput. 13, 163–167. 10.1023/A:1023260610025

[B33] McCullagh P. Nelder J. A. (1989). Generalized Linear Models, 2nd Edn London: Chapman & Hall.

[B34] Montgomery D. C. (1999). Experimental design for product and process design and development. J. R. Stat. Soc. Series D 48, 159–177. 10.1111/1467-9884.00179

[B35] Nnadi C. U. Mimiko O. A. Mccurtis H. L. Cadet L. (2005). Neuropsychiatric effects of cocaine use disorders. J. Natl. Med. Assoc. 97, 1504–1515. 16334497 PMC2594897

[B36] Nutt D. King L. A. Saulsbury W. Blakemore C. (2007). Development of a rational scale to assess the harm of drugs of potential misuse. Lancet 369, 1047–1053. 10.1016/S0140-6736(07)60464-4 17382831

[B37] Olesen J. Gustavsson A. Svensson M. Wittchen H. U. Jonsson B. (2012). The economic cost of brain disorders in Europe. Eur. J. Neurol. 19, 155–162. 10.1111/j.1468-1331.2011.03590.x 22175760

[B38] Panksepp J. (1998). Affective Neuroscience: The Foundations of Human and Animal Emotions. Oxford: Oxford University Press.

[B39] Pennings E. J. M. Leccese A. P. De Wolff F. A. (2002). Effects of concurrent use of alcohol and cocaine. Addiction 97, 773–783. 10.1046/j.1360-0443.2002.00158.x 12133112

[B40] Platt M. L. Huettel S. A. (2008). Risky business: the neuroeconomics of decision making under uncertainty. Nat. Neurosci. 11, 398–403. 10.1038/nn2062 18368046 PMC3065064

[B41] Preller K. H. Hulka L. M. Vonmoos M. Jenni D. Baumgartner M. R. Seifritz E.. (2014). Impaired emotional empathy and related social network deficits in cocaine users. Addict. Biol. 19, 452–466. 10.1111/adb.12070 23800218

[B42] Preller K. H. Ingold N. Hulka L. M. Vonmoos M. Jenni D. Baumgartner M. R.. (2013). Increased sensorimotor gating in recreational and dependent cocaine users is modulated by craving and attention-deficit/hyperactivity disorder symptoms. Biol. Psychiatry 73, 225–234. 10.1016/j.biopsych.2012.08.003 22959126

[B43] Quednow B. B. Herdener M. (2016). Human pharmacology for addiction medicine: from evidence to clinical recommendations. Prog. Brain Res. 224, 227–250. 10.1016/bs.pbr.2015.07.017 26822361

[B44] Quednow B. B. Kuhn K. U. Hoenig K. Maier W. Wagner M. (2004). Prepulse inhibition and habituation of acoustic startle response in male MDMA (‘ecstasy’) users, cannabis users, and healthy controls. Neuropsychopharmacology 29, 982–990. 10.1038/sj.npp.1300396 14970829

[B45] Ritz M. C. Lamb R. J. Goldberg S. R. Kuhar M. J. (1987). Cocaine receptors on dopamine transporters are related to self-administration of cocaine. Science 237, 1219–1223. 10.1126/science.2820058 2820058

[B46] Robinson T. E. Berridge K. C. (2001). Incentive-sensitization and addiction. Addiction 96, 103–114. 10.1046/j.1360-0443.2001.9611038.x 11177523

[B47] Robinson T. E. Kolb B. (2004). Structural plasticity associated with exposure to drugs of abuse. Neuropharmacology 47(Suppl. 1), 33–46. 10.1016/j.neuropharm.2004.06.025 15464124

[B48] Roesler M. Retz W. Retz-Junginger P. Thome J. Supprian T. Nissen T. (2004). Instrumente zur diagnostik der Aufmerksamkeitsdefizit-/Hyperaktivitätsstörung (ADHS) im Erwachsenenalter. Nervenarzt 75, 888–895. 10.1007/s00115-003-1622-2 15378249

[B49] Schubert R. Brown M. (1999). Financial decision-making: are women really more risk-averse? Am. Econ. Rev. 89, 386–391.

[B50] Schultz W. (2010). Dopamine signals for reward value and risk: basic and recent data. Behav. Brain Funct. 6:24. 10.1186/1744-9081-6-24 20416052 PMC2876988

[B51] Schultz W. (2015). Neuronal reward and decision signals: from theories to data. Physiol. Rev. 95, 853–951. 10.1152/physrev.00023.2014 26109341 PMC4491543

[B52] Searle S. R. (1987). Linear Models for Unbalanced Data. New York, NY: John Wiley & Sons.

[B53] Shafir E. (2008). Desicion making, in MIT Encyclopedia of the Cognitive Sciences, eds Wilson R. A. Keil F. C. (Cambridge, MA: MIT Press), 220–223.

[B54] Soar K. Mason C. Potton A. Dawkins L. (2012). Neuropsychological effects associated with recreational cocaine use. Psychopharmacology (Berl). 222, 633–643. 10.1007/s00213-012-2666-4 22374254

[B55] Sribney B. (2011). What are Some of the Problems with Stepwise Regression [Online]. StataCorp. Available online at: http://www.stata.com/support/faqs/statistics/stepwise-regression-problems/ (Accessed March 13, 2016).

[B56] Strauss E. Sherman E. M. S. Spreen O. A. (2006). Compendium of Neuropsychological Tests: Administration, Norms, and Commentary. Oxford, UK: Oxford University Press.

[B57] Sussner B. D. Smelson D. A. Rodrigues S. Kline A. Losonczy M. Ziedonis D. (2006). The validity and reliability of a brief measure of cocaine craving. Drug Alcohol Depend. 83, 233–237. 10.1016/j.drugalcdep.2005.11.022 16384655

[B58] Tversky K. (1992). Advances in prospect theory: cumulative representation of uncertainty. J. Risk Uncertain. 5, 297–323. 10.1007/BF00122574

[B59] Vadhan N. P. Hart C. L. Haney M. Van Gorp W. G. Foltin R. W. (2009). Decision-making in long-term cocaine users: effects of a cash monetary contingency on Gambling task performance. Drug Alcohol Depend. 102, 95–101. 10.1016/j.drugalcdep.2009.02.003 19346083 PMC2694492

[B60] Verdejo-Garcia A. Benbrookb A. Funderburkc F. Davidb P. Cadetd J. L. Bolla K. (2007). The differential relationship between cocaine use and marijuana use on decision-making performance over repeat testing with the Iowa Gambling Task. Drug Alcohol Depend. 90, 2–11. 10.1016/j.drugalcdep.2007.02.004 17367959 PMC1986840

[B61] Vonmoos M. Hulka L. M. Preller K. H. Jenni D. Baumgartner M. R. Stohler R.. (2013a). Cognitive dysfunctions in recreational and dependent cocaine users: role of attention-deficit hyperactivity disorder, craving and early age at onset. Br. J. Psychiatry 203, 35–43. 10.1192/bjp.bp.112.118091 23703315

[B62] Vonmoos M. Hulka L. M. Preller K. H. Jenni D. Schulz C. Baumgartner M. R.. (2013b). Differences in self-reported and behavioral measures of impulsivity in recreational and dependent cocaine users. Drug Alcohol Depend. 133, 61–70. 10.1016/j.drugalcdep.2013.05.032 23806872

[B63] Vonmoos M. Hulka L. M. Preller K. H. Minder F. Baumgartner M. R. Quednow B. B. (2014). Cognitive impairment in cocaine users is drug-induced but partially reversible: evidence from a longitudinal study. Neuropsychopharmacology 39, 2200–2210. 10.1038/npp.2014.71 24651468 PMC4104339

[B64] Wagner F. A. Anthony J. C. (2002). From first drug use to drug dependence; developmental periods of risk for dependence upon marijuana, cocaine, and alcohol. Neuropsychopharmacology 26, 479–488. 10.1016/S0893-133X(01)00367-0 11927172

[B65] Wechsler D. A. (1997). Wechsler Memory Scale, 3rd Edn. Manual. San Antonio, TX: Psychological Corporation.

[B66] Zack M. Poulos C. X. (2009). Parallel roles for dopamine in pathological gambling and psychostimulant addiction. Curr. Drug Abuse Rev. 2, 11–25. 10.2174/1874473710902010011 19630734

